# Caregiver burden among parents of school-age children with asthma: a cross-sectional study

**DOI:** 10.3389/fpubh.2024.1368519

**Published:** 2024-06-05

**Authors:** Fang Yang, Jingru Zhou, Hongying Xiao, Xia Wu, Yingjuan Cui, Houqiang Huang, Silin Zheng, Huawei Li

**Affiliations:** ^1^Department of Pediatrics, Deyang People's Hospital, Deyang, Sichuan, China; ^2^Department of Surgery, Affiliated Hospital of North Sichuan Medical College, Nanchong, Sichuan, China; ^3^Nursing Department, The Affiliated Hospital of Southwest Medical University, Luzhou, Sichuan, China; ^4^Nephrology Department, Affiliated Hospital of Nanjing University of Chinese Medicine, Nanjing, Jiangsu, China

**Keywords:** school-age, asthma, parents, caregivers, caregiver burden

## Abstract

**Objective:**

To investigate the caregiver burden of parents of school-age children with asthma and analyze the factors influencing their caregiver burden.

**Methods:**

A convenience sampling method was used to select 366 parents of school-age children with asthma who visited the outpatient departments of three tertiary hospitals in Sichuan Province, China, from January 2021 to July 2021. A general information questionnaire and the Caregiver Burden Inventory (CBI) were used to assess the current caregiver burden and analyze the influencing factors.

**Results:**

The caregiver burden score of parents of school-age children with asthma was 27 (17, 39), with 40.43% of parents experiencing moderate to high levels of burden. Detailed results of univariate analysis showed that there were significant differences in caregiver burden scores based on parents’ gender, highest education level, number of children, occupation, family history of asthma, monthly family income, annual medical expenses for the child, child’s gender, whether the child had undergone lung function tests, number of emergency visits due to asthma exacerbation in the past 3 months, and whether the child had missed school due to asthma exacerbation in the past 3 months (*p* < 0.1). Detailed results of multivariate analysis showed that parents’ gender, occupation, family history of asthma, monthly family income, annual medical expenses for the child, number of emergency visits due to asthma exacerbation in the past 3 months, and whether the child had missed school due to asthma exacerbation in the past 3 months were independent risk factors for caregiver burden in parents of school-age children with asthma (*p* < 0.05).

**Conclusion:**

Parents of school-age children with asthma experience a certain level of caregiver burden, with over one-third of parents experiencing moderate to high levels of burden. Being a mother, being a worker, having no family history of asthma, having low monthly family income, having high annual medical expenses for the child, having frequent emergency visits due to asthma exacerbation in the past 3 months, and having missed school due to asthma exacerbation in the past 3 months are independent risk factors for caregiver burden in parents of school-age children with asthma, healthcare providers should develop feasible coping strategies, such as paying attention to caregivers’ psychological condition to reduce the burden of caring for parents of school-age children with asthma. The entire society should also make efforts in improving social support and strengthening healthcare coverage in order to achieve the aforementioned goals.

## 1 Introduction

Asthma is one of the most common chronic respiratory disease worldwide (1), affecting over 300 million people globally (2). The prevalence of asthma is approximately 5.00–10.00% in adults and 20.00% in children (3), with school-age children (6–14 years old) being a high-risk group for asthma (4). Numerous studies have shown that asthma has various and significant impacts on the health, learning, and social interactions of affected children (5, 6), as well as on the daily life, physical and mental health of their primary caregivers and families (7, 8).

Caregiver burden refers to the negative physiological, psychological, and economic stimuli and pressures experienced by caregivers during the caregiving process (9, 10). As the primary caregivers of school-age children with asthma (11), parents bear a series of burdens associated with the disease. Firstly, asthma is a prominent health issue among children (12, 13). Global studies have shown that only a small proportion of children with asthma have good disease control, with the situation being worse in China (14), especially among school-age children (15). Poor asthma control can lead to decreased lung function during the crucial period of lung development and function transition in childhood (16). Additionally, due to the ongoing growth and development of children, recurrent asthma exacerbations can result in growth retardation and reduced quality of life (17), and become important reasons for school absenteeism among school-age children (18, 19). Moreover, compared to their peers, school-age children with asthma are more likely to experience behavioral problems, learning difficulties, anxiety, and other issues (20). Secondly, there is currently no effective cure for asthma, and long-term, standardized, scientific, and effective disease management is necessary (21). In developed countries, the financial burden of asthma is relatively high, accounting for 1.00–2.00% of healthcare expenditures in countries with available medical expenditure data (22, 23). According to data from the US National Medical Expenditure Panel Survey from 2007 to 2013, the total annual medical expenses for school-age children with asthma amounted to 5.92 billion US dollars (22). In China, 37.80% of families with children with asthma bear annual medical expenses exceeding 10,000 yuan, and 27.60% of children with asthma have annual medical expenses exceeding 5,000 yuan (24). Thus, asthma poses a significant economic burden on both families and society (19, 25). Studies have also found that parents of children with asthma experience varying degrees of anxiety and depression during the long-term caregiving process (26). 81.52% of caregivers reported high parenting stress and psychological distress (27), 67.57% of caregivers experienced varying degrees of depression, and 29.00% of caregivers were diagnosed with post-traumatic stress disorder (27, 28).

In summary, asthma has various and significant impacts on school-age children and their parents. It is important to pay attention to the caregiver burden of parents of school-age children with asthma. However, current research mainly focuses on the caregiver burden of primary caregivers of children aged 0–14 with asthma (29–32), and there is a lack of studies specifically examining the caregiver burden of parents of school-age children (6–14 years old) with asthma. Therefore, this study aims to investigate the caregiver burden of parents of school-age children with asthma through a cross-sectional survey and analyze the influencing factors. The findings will provide practical evidence for formulating targeted strategies to alleviate the caregiver burden of parents of school-age children with asthma.

## 2 Materials and methods

### 2.1 Survey subjects

Convenience sampling method was used to select parents of school-age children with asthma who visited the pediatric outpatient departments of three tertiary hospitals in Sichuan Province, China from January 2021 to July 2021 as the research subjects. Inclusion criteria: ① Parents of children diagnosed with asthma according to the diagnostic criteria of the Chinese Medical Association’s Pediatric Branch Respiratory Group’s “Diagnosis and Treatment Guidelines for Children’s Bronchial Asthma” (33); ② Parents of children aged 6–14 years (34); ③ The parents of the children have the ability to think and express themselves in language and a certain level of reading and comprehension skills; ④ Willing to participate in this study and sign an informed consent form. Exclusion criteria: ① Children with organic diseases, mental illnesses, or other severe chronic diseases other than asthma; ② Parents with a history of mental illness or severe chronic diseases who are unable to complete the questionnaire independently; ③ Children and their parents who have experienced significant trauma in the past 3 months. This part of the study includes 9 items on general information of parents, 6 items on general information of children, and 24 items on caregiver burden inventory, for a total of 39 analyzed variables. Sample size calculation adopted Li Zheng’s rough estimation method (35), with a sample size of 5–10 times the number of variables, considering a 20.00% increase in sample size for invalid questionnaires. The calculated sample size for this study was 234–468 people, with 380 included samples and 366 valid questionnaires, resulting in a questionnaire validity rate of 96.30%. This study was approved by the Ethical Committee of Deyang People’s Hospital (No. 2021-04-21-K01), the Ethical Committee of Affiliated Hospital of Southwest Medical University (No. KY2021223), and the Ethical Committee of Affiliated Hospital of North Sichuan Medical College (No. 2022ER021-1).

### 2.2 Survey tools

#### 2.2.1 General information questionnaire

Including general information questionnaires for children and parents. The demographic characteristics of children include age, gender, education method, course of disease, whether pulmonary function tests were performed, number of outpatient visits due to worsening of asthma in the past 3 months, number of school absenteeism days, etc. The demographic characteristics of parents include age, gender, education level, occupation, marital status, number of children, monthly income, smoking status, child’s medical expenses, family history of asthma, etc.

#### 2.2.2 Caregiver Burden Inventory (CBI)

The Caregiver Burden Inventory (CBI), developed by Novak and Guest (36) in 1989, is widely used in domestic and international caregiver research to assess the subjective burden of caregivers during the caregiving process. The CBI questionnaire used in this study was translated and revised by Chinese scholar Yue Peng (37) in 2006. The Chinese version of the questionnaire consists of 5 dimensions: time-dependent burden (items 1–5), developmental burden (items 6–10), physical burden (items 11–14), social burden (items 15–18), and emotional burden (items 19–24), with a total of 24 items. The questionnaire uses a 5-point scoring standard, with scores ranging from 0 to 4. A score of 0 indicates “never” and a score of 4 indicates “always.” The total score ranges from 0 to 96, with scores of 0 to 32 indicating mild burden, 33 to 64 indicating moderate burden, and scores above 65 indicating severe burden. The internal consistency reliability of the questionnaire is 0.92, and the Cronbach’s alpha coefficients of each dimension range from 0.68 to 0.93. In this study, the internal consistency reliability of the questionnaire was 0.85, and the Cronbach’s alpha coefficients of each dimension were 0.87.

#### 2.2.3 Data collection and quality control methods

Convenience sampling method was used in this study to strictly select survey subjects according to the inclusion and exclusion criteria. In order to achieve homogenization of the study, the research team members received unified training. Before completing the questionnaire, the researchers explain it to the research subjects and obtain their written informed consent. If the research subjects had any questions during the process of filling out the questionnaire, the researchers provided on-site explanations. After completing the questionnaire, it was collected on the spot to reduce data loss. The entire data collection process lasted for 7 months.

### 2.3 Statistical analysis

A database was established using Excel 2007 software, and the data was organized. SPSS 25.0 statistical software was used for data analysis. If quantitative data followed a normal distribution, it was described using mean ± standard deviation (SD); otherwise, median (M) and interquartile range (P25, P75) were used. Qualitative data were described using frequencies and percentages. Single-factor analysis of variance (Mann–Whitney U test) or multiple independent samples rank sum test (Kruskal-Wallis H test) were used to analyze the association between parents’ and individual factors and caregiver burden scores, depending on the number of influencing factors. Multiple logistic regression analysis was used to analyze the factors associated with high caregiver burden. The 70th and 80th percentiles of the total burden score and each dimension score were used as the cut-off points for high burden, and the variables with *p* < 0.10 in the univariate analysis were included in the model, with the model fit using the Forward: LR method. A significance level of *p* < 0.05 was considered statistically significant.

## 3 Results

A total of 380 questionnaires were distributed and collected, with 366 valid questionnaires and 14 invalid questionnaires (6 questionnaires had too many missing answers, exceeding 4 items; 5 questionnaires had a regular pattern of selected options; 3 questionnaires were filled out by the same caregiver). The questionnaire validity rate was 96.30%.

### 3.1 General information

#### 3.1.1 General information of parents of children with asthma

The average age of parents was (34.4 ± 5.1) years. There were 79 males (21.60%) and 287 females (78.40%). In terms of education level, 53 had junior high school education or below (14.40%), 113 had high school or technical secondary school education (30.90%), 118 had college education (32.20%), and 82 had master’s degree or above (22.50%). In terms of the number of children, 233 had 1 child (63.70%) and 133 had 2 or more children (36.30%) (see Table 1 for details).

**Table 1:** Demographic characteristics of children’s parents (*n* = 366).

Item	*N*	Constituent ratio (%)
Parents’ gender		
Male	79	21.60
Female	287	78.40
Parents’ age (year)		
<30	48	13.10
30~	259	70.80
≥40	59	16.10
Parents’ highest education level		
Junior high school education or below	53	14.40
High school or technical secondary school education	113	30.90
College education	118	32.20
Master’s degree or above	82	22.50
Number of children		
1 child	233	63.70
2 or more children	133	36.30
Occupation		
Worker	52	14.20
Farmer	63	17.20
Administrative worker	44	12.00
Service industry	129	35.20
Private business owner	78	21.40
Someone smoking at home		
Yes	199	54.40
No	167	45.60
Family history of asthma		
Yes	55	15.00
No	311	85.00
Monthly family income (CNY)		
3,000 ~ 4,999	128	35.00
5,000 ~ 9,999	142	38.80
≥10,000	96	26.20
Annual medical expenses of the child (CNY)		
<3,000	122	33.30
3,000 ~ 4,999	154	42.10
≥5,000	90	24.60

#### 3.1.2 Demographic characteristics of children

The average age of children was (7.7 ± 1.7) years. There were 203 boys (55.50%) and 163 girls (39.30%). Course of illness≤1 year 144 (39.30%), 1 ~ 2 year 141 (38.50%), ≥3 year 81 (22.10%) (see Table 2 for details).

**Table 2:** Demographic characteristics of children (*n* = 366).

Item	*N*	Constituent ratio (%)
Child’s gender		
Boy	203	55.50
Girl	163	44.50
Child’s age (year)		
6~	121	33.10
7~	94	25.70
8~	73	19.90
≥9	78	21.30
Child’s illness course (year)		
≤1	144	39.30
1 ~ 2	141	38.50
≥3	81	22.10
Whether the child had undergone lung function tests		
Yes	340	92.90
No	26	7.10
Number of emergency visits due to asthma exacerbation in the past 3 months (time)		
0	112	30.60
1	95	26.00
2	69	18.90
≥3	90	24.50
Whether the child had missed school due to asthma exacerbation in the past 3 months		
No	198	54.10
Yes	168	45.90

### 3.2 The caregiver burden score of parents of school-age children with asthma

#### 3.2.1 The caregiver burden score

The total caregiver burden score was 27 (17, 39) points, 148 parents with school-age children with asthma is ≥32 points, indicating that 40.43% of parents with school-age children with asthma had a moderate to high level of caregiver burden. Among them, the score for time-dependent burden dimension was 9 (6, 13) points, the score for development-restricted burden dimension was 7 (4, 11) points, the score for physical burden dimension was 5 (2, 8) points, and the score for social burden dimension was 3 (1, 6) points (see Table 3 for details).

**Table 3:** The results of caregiver burden (*n* = 366).

Dimension	Scoring range	Min	P_25_	P_50_	P_75_	Max	Sorting
Time-dependence	0 ~ 20	0	6	9	13	20	1
Developmental	0 ~ 20	0	4	7	11	20	2
Physical	0 ~ 16	0	2	5	8	16	3
Social	0 ~ 16	0	1	3	6	16	4
Emotional	0 ~ 24	0	0	1	5	24	5
Caregiver burden	0 ~ 96	2	17	27	39	96	

#### 3.2.2 Univariate analysis of the caregiver burden

There were significant differences in caregiver burden scores based on parents’ gender, highest education level, number of children, occupation, family history of asthma, monthly family income, annual medical expenses for the child, child’s gender, whether the child had undergone lung function tests, number of emergency visits due to asthma exacerbation in the past 3 months, and whether the child had missed school due to asthma exacerbation in the past 3 months (*p* < 0.1) (see Table 4 for details). The original analysis results of detailed data can be found in supplements.

**Table 4:** Univariate analysis variable screening results.

Variable	Time-dependence	Developmental	Physical	Social	Emotional	Caregiver burden
Parents’ gender	√	√	√	—	—	√
Parents’ age	—	—	—	—	—	—
Parents’ highest education level	—	—	√	—	—	—
Number of children	√	—	—	—	√	—
Parents’ occupation	√	—	√	√	—	√
Someone smoking at home	—	—	—	—	—	—
Family history of asthma	—	√	—	—	√	√
Family monthly income	—	—	√	—	—	—
Annual medical expenses of the child	—	√	√	—	—	√
Child’s gender	√	—	—	—	—	—
Child’s age	—	—	—	—	—	—
Child’s illness course	—	—	—	—	—	—
Whether the child had undergone lung function tests	—	—	—	—	√	—
Number of emergency visits due to asthma exacerbation in the past 3 months	√	√	√	√	√	√
Whether the child had missed school due to asthma exacerbation in the past 3 months	√	√	√	√	√	√

“√” represents the factors that have an impact on CBI dimensions and overall burden, with *p* < 0.10; “—” represents *p* > 0.10.

#### 3.2.3 Multivariate analysis of the caregiver burden

Based on the total burden score and the scores of each dimension at the 70th percentile (P70) and 80th percentile (P80), a burden score greater than P70 or P80 is defined as an excessive caregiver burden. Logistic regression models were fitted with factors with a *p*-value less than 0.10 in the single-factor analysis as independent variables, using whether the caregiver burden is excessive as the dependent variable. The corresponding models are referred to as model1 and model2. The P70 and P80 for the total score and scores of each dimension are shown in Table 5. Variable assignments can be found in Table 6.

**Table 5:** Total caregiver burden score and P70 and P80 of each dimension score.

Percentile	Caregiver burden	Time-dependence	Developmental	Physical	Social	Emotional
P_70_	36	12	10	8	5	3
P_80_	43	14	12	9	7	6

**Table 6:** Multivariate logistic regression analysis variable assignment table.

Independent variable	Assignment			
Dependent variable				
Is the burden too high	1 = yes	0 = normal		
Independent variable				
Parents’ gender	1 = male	2 = female		
Parents’ highest education level	1 = junior high school education or below	2 = high school or technical secondary school education	3 = college education	4 = master’s degree or above
Number of children	1 = 1 child	2 = 2 or more children		
Parents’ occupation	1 = worker	2 = farmer	3 = administrative worker	
	4 = service industry	5 = private business owner		
Family history of asthma	0 = no	1 = yes		
Monthly family income	1 = 3,000 ~ 4,999 (CNY)	2 = 5,000 ~ 9,999 (CNY)	3 = 10,000 or above (CNY)	
Annual medical expenses for the child	1 = 3,000 or below (CNY)	2 = 3,000 ~ 4,999 (CNY)	3 = 5,000 or above (CNY)	
Child’s gender	1 = boy	2 = girl		
Whether the child had undergone lung function tests	0 = no	1 = yes		
Number of emergency visits due to asthma exacerbation in the past 3 months	0 = time	1 = 1time	2 = 2 times	3 = 3 times or above
Whether the child had missed school due to asthma exacerbation in the past 3 months	0 = no	1 = yes		

All multi-class variables are included in dummy variable form.

Multiple factor logistic regression analysis shows: Parents’ gender (female), occupation (worker), family history of asthma (no family history of asthma), monthly family income (low monthly family income), annual medical expenses for the child (high annual medical expenses for child), number of emergency visits due to asthma exacerbation in the past 3 months (frequent emergency visits due to asthma exacerbation in the past 3 months), and whether the child had missed school due to asthma exacerbation in the past 3 months (missed school due to asthma exacerbation in the past 3 months) were independent risk factors for caregiver burden in parents of school-age children with asthma (*p* < 0.05) (see Table 7). The original analysis results of detailed data can be found in supplements.

**Table 7:** Multivariate logistic regression analysis results summary.

Independent variable	Time-dependence	Developmental	Physical	Social	Emotional	Caregiver burden
Parents’ gender	√	—	—	—	—	—
Parents’ occupation	—	—	√	√	—	√
Family history of asthma	—	—	—	—	√	—
Monthly family income	—	—	√	—	—	—
Annual medical expenses for the child	—	—	√	—	—	√
Number of emergency visits due to asthma exacerbation in the past 3 months	—	√	√	—	—	√
Whether the child had missed school due to asthma exacerbation in the past 3 months	√	—	—	√	√	—

“√” represents the factors that influence the burden of CBI in each dimension and overall, i.e., *p* < 0.05; “—” represents *p* > 0.05.

## 4 Discussion

The total burden of care score was 27 (17, 39), and among 366 parents, 148 scored ≥32, indicating that over one-third of parents of school-age children with asthma experience a moderate to high level of caregiver burden in our study. The dimensions of caregiver burden were ranked as follows: time-dependence burden, developmental restriction burden, physical burden, social burden, and emotional burden. These rankings were consistent with previous studies (38), parents of school-age children with asthma face long-term uncertainty related to their child’s worsening condition. Additionally, they devote significant time and effort to children with asthma, leading to physical and mental strain. The anxiety related to time constraints and the compression of parents’ working hours by caregiver responsibilities exacerbate the sense of time-dependence burden.

Gender was found to be a factor influencing the time-dependence burden dimension, with mothers being at a higher risk of experiencing excessive burden compared to fathers. Several studies have shown that female caregivers bear a greater burden during long-term care for diseases (39, 40). This can be attributed to the fact that mothers are often the primary caregivers for children within the family (41, 42), investing more time and energy in their care. Furthermore, women are more prone to experiencing negative emotions such as stress and anxiety due to physiological and adaptability differences, resulting in increased physical and emotional burden (38, 42). Support systems refers to the external resources that individuals can utilize, mainly referring to the material support or psychological help obtained from family, colleagues, and friends in their social life (43). Researches have shown solid support system can enhance caregivers’ ability to cope with challenges (44). It is recommended that family members, especially spouses, understand and support each other, taking turns in providing care and companionship for the child (38). Two studies have shown that the caregivers’ mental status affects the caregivers’ burden level, and psychological intervention can reduce caregivers’ sense of stress, anxiety, and loneliness, as well as reduce caregivers’ burden (45, 46). So, healthcare professionals should pay attention to the psychological well-being of mothers and provide necessary psychological interventions (47) such as cognitive-behavioral therapy, family therapy, motivational interviewing, and problem-solving therapy to alleviate the physical and mental stress experienced by mothers as primary caregivers.

Family monthly income and annual medical expenses of the child were found to be factors influencing the physical burden dimension. Wang Jing et al. (48) also identified economic issues as the main influencing factors of caregiver burden among family caregivers. This can be explained by the fact that asthma, as a chronic disease, requires financial support for its treatment and care. In order to ensure the continuity and effectiveness of their child’s treatment, parents often choose to give up their jobs to take care of their child full-time (49), or they may increase their work hours or take on multiple jobs to earn more money (50). However, they still need to balance the responsibilities of supporting older adult family members and caring for their sick child, often neglecting their own health (38). This leads to excessive physical burden. It is suggested that diversified strategies be implemented at the national level, such as providing more job opportunities or flexible working hours for caregivers (44), establishing specialized medical insurance programs for children with asthma, promoting affordable medications (51), and implementing home nebulization therapy (50), in order to alleviate the economic pressure on caregivers.

The number of emergency visits due to asthma exacerbation in the past 3 months and the child’s absenteeism due to asthma were found to be factors influencing multiple dimensions of caregiver burden. Several studies have indicated that recent stressful events have a negative impact on individuals’ quality of life (52), which is consistent with the findings of Jiang Di (53). In this study, the stressful events experienced by caregivers were “the number of emergency visits due to asthma exacerbation in the past three months” and “the number of school absences due to asthma in the past three months.” This can be attributed to the greater caregiver difficulties faced by parents of children with asthma compared to parents of healthy children. Asthma exacerbations lead to frequent leaves from work to visit hospitals, deal with delays in the child’s education, and handle school absences, which inevitably sacrifices the caregiver’s rest time and social activities (54). Hospitals should pay more attention to children with frequent hospitalizations and poor disease control, as well as their family caregivers, providing targeted advice and recommendations to reduce the frequency of disease relapses (30). Additionally, weekend specialist asthma clinics should be increased (55), and the development of internet hospitals should be accelerated to expand online services (56). It is also important to establish communication platforms for caregivers, such as WeChat or QQ groups, organizing parent–child activities, and peer support meetings (44, 57), to facilitate the sharing of caregiver experiences and promote better care for the child.

## 5 Conclusion

This study reveals that parents of school-age children with asthma experience a certain level of caregiver burden, with over one-third of parents experiencing moderate to high levels of burden. Being a mother, being a worker, having no family history of asthma, having low monthly family income, having high annual medical expenses for the child, having frequent emergency visits due to asthma exacerbation in the past 3 months, and having missed school due to asthma exacerbation in the past 3 months are independent risk factors for caregiver burden in parents of school-age children with asthma, healthcare providers should develop feasible coping strategies, such as paying attention to caregivers’ psychological condition to reduce the burden of caring for parents of school-age children with asthma. The entire society should also make efforts in improving social support and strengthening healthcare coverage in order to achieve the aforementioned goals.

## 6 Strengths and limitations

The impact of asthma on school-age children and their parents is multifaceted and significant. As the primary caregivers of school-age children with asthma, parents bear a series of burdens brought about by the disease. However, current research primarily focuses on the caregiver burden of parents of children with asthma aged 0–14. This study is the first to investigate the caregiver burden of parents of school-age children (6–14 years old) with asthma in China, and of course, this study has limitations such as a limited range of research tools. In the future, more research tools should be used and more variables should be included to further improve the research results.

## Data availability statement

The original contributions presented in the study are included in the article/Supplementary materials, further inquiries can be directed to the corresponding authors.

## Ethics statement

This study was approved by the Ethical Committee of Deyang People’s Hospital (No. 2021-04-21-K01), the Ethical Committee of Affiliated Hospital of Southwest Medical University (No. KY2021223), and the Ethical Committee of Affiliated Hospital of North Sichuan Medical College (No. 2022ER021-1). The studies were conducted in accordance with the local legislation and institutional requirements. The participants provided their written informed consent to participate in this study.

## Author contributions

FY: Supervision, Project administration, Data curation, Writing – review & editing, Writing – original draft. JZ: Software, Methodology, Data curation, Writing – review & editing. HX: Investigation, Writing – review & editing, Data curation. XW: Writing – review & editing, Investigation, Data curation. YC: Writing – review & editing, Investigation, Data curation. HH: Writing – review & editing, Investigation, Data curation. SZ: Writing – original draft, Supervision, Project administration, Formal analysis, Writing – review & editing. HL: Project administration, Writing – review & editing, Supervision, Formal analysis, Conceptualization.

## References

[1] Shang B Li X Xu Y Ren W Wang J Xing C. Clinical characteristics and economic burden of asthma in China: a multicenter retrospective study. Iran J Allergy Asthma Immunol. (2023) 22:290–8. doi: 10.18502/ijaai.v22i3.13057, PMID: 37524665

[2] Global, regional, and national deaths, prevalence, disability-adjusted life years, and years lived with disability for chronic obstructive pulmonary disease and asthma, 1990-2015: a systematic analysis for the global burden of disease study 2015. Lancet Respir Med. (2017). doi: 10.1016/S2213-2600(17)30293-X PMC5573769 28822787

[3] Bagnasco D Paggiaro P Latorre M Folli C Testino E Bassi A. Severe asthma: one disease and multiple definitions. World Allergy Organ J. (2021) 14:100606. doi: 10.1016/j.waojou.2021.100606, PMID: 34871335 PMC8609160

[4] Mou JH Shao MJ Liu CH Sha L Zhu WJ Li S. Comparison of food allergy among children with bronchial asthma and children without bronchial asthma in Chinese cities. Chinese Clin J Pract Pediatr. (2018) 33:684–7. doi: 10.3760/cma.j.issn.2095-428X.2018.09.008

[5] Xie T Zhong LL Huang H Lin XJ Xiao LG Pen L. Respiratory tract of 225 children with acute attack of bronchial asthma physical examination and clinical characteristics analysis of pathogens. Chin J Contemp Pediatr. (2020) 20:1198–203. doi: 10.7499/j.issn.1008-8830.2006024 PMC7666394 33172555

[6] Zhao XQ Qin QZ Jing W Zhang X. Investigation on hospital stay and family economic burden of children with severe asthma under 14 years old. Public Health Prevent Med. (2021) 32:96–9. doi: 10.3969/j.issn.1006-2483.2021.04.022

[7] Gibson-Young LM Arolan KJ Wegucki LS Lang JE Norris CL. Interviens with aregivers during acute asthma hepitalisatins. J Ashma. (2020) 57:778–86. doi: 10.1080/02770903.2019.1602875 31025890

[8] Foronda CL Kelley CN Nadeau C Prather SL Lewis-Pierre L Sarik DA. P'sychobgieal and socioecmnomie burdens faed by family cargivers of children with asthm; an integative review. J Pediatr Health Care. (2020) 34:366–76. doi: 10.1016/j.pedhc.2020.02.003, PMID: 32299726

[9] Liu S Li C Shi Z Wang X Zhou Y Liu S. Caregiver burden and prevalence of depression, anxiety and sleep disturbances in Alzheimer's disease caregivers in China. J Clin Nurs. (2017) 26:1291–300. doi: 10.1111/jocn.13601, PMID: 27681477

[10] Liu SJ Zhang YH Tang MW Cheng Y. The impact of stigma on the caregiver burden of patients with schizophrenia. Chin J Nurs. (2019) 56:239–44. doi: 10.3761/j.issn.0254-1769.2021.02.013

[11] Hua K Mj Y Sd X. The mediating effect of primary caregivers' self-efficacy on the influence of coping style on quality of life in children with asthma. Modern Clinical Nursing. (2021) 20:14–8. doi: 10.3969/j.issn.1671-8283.2021.9.03

[12] Caffrey Osvald E Bower H Lundholm C Larsson H Brew BK Almqvist C. Asthma and all-cause mortality in children and young adults: a population-based study. Thorax. (2020) 75:1040–6. doi: 10.1136/thoraxjnl-2020-214655, PMID: 32963117 PMC7677462

[13] Asher MI Rutter CE Bissell K Chiang CY El Sony A Ellwood E. Global asthma network phase I study group. Worldwide trends in the burden of asthma symptoms in school-aged children: global asthma network phase I cross-sectional study. Lancet. (2021) 398:1569–80. doi: 10.1016/S0140-6736(21)01450-1, PMID: 34755626 PMC8573635

[14] Lin J Wang W Tang H Huo J Gu Y Liu R. Asthma management using the Mobileasthma evaluation and management system in China. Allergy, Asthma Immunol Res. (2022) 14:85–98. doi: 10.4168/aair.2022.14.1.85, PMID: 34983109 PMC8724822

[15] Jia Y Wang H Zhang Z Wang J Yi M Chen O. Parenting style and child asthma control in families of school-age children with asthma: the mediating effects of children's general self-efficacy and medication adherence. J Pediatr Nurs. (2023) 73:e293–301. doi: 10.1016/j.pedn.2023.09.025, PMID: 37805379

[16] Zheng YJ Chen JH Shen KL. The childhood origin of adult respiratory diseases. Chin J Pract Pediatr. (2017) 32:1201–4. doi: 10.3760/cma.j.issn.2095-428X.2017.16.001

[17] Luo P Bai YQ Liu H Long D. Investigation on self-perceived burden of children with bronchial asthma aged 3-6 years and analysis of influencing factors. South China Prevent Med. (2019) 46:22–5.

[18] Pijnenburg MW Fleming L. Advances in understanding and reducing the burden of severe asthma in children. Lancet Respir Med. (2020) 8:1032–44. doi: 10.1016/S2213-2600(20)30399-4, PMID: 32910897

[19] Prather SL Foronda CL Kelley CN Nadeau C Prather K. Barriers and facilitators of asthma management as experienced by African American caregivers of children with asthma: an integrative review. J Pediatr Nurs. (2020) 55:40–74. doi: 10.1016/j.pedn.2020.06.012, PMID: 32653828

[20] He HM Chen Q Wang GN. Effect of quality nursing program "asthma home" on out-of-hospital follow-up intervention of children with asthma. J Guangxi Med Univ. (2019) 36:174–7.

[21] Knafl KA Havill NL Leeman J Fleming L Crandell JL Sandelowski M. The nature of family engagement in interventions for children with chronic conditions. West J Murs Res. (2017) 39:690–723. doi: 10.1177/0193945916664700, PMID: 27596106 PMC5581947

[22] Serebrisky D. Wiznia A Pediatric asthma: a global epidemic. Ann Glob Health. (2019) 85:6. doi: 10.5334/aogh.2416, PMID: 30741507 PMC7052318

[23] Trikamjee T Comberiati R. Peter」pediatric asthma in developing countries:challenges and_ future directions. Curr Opin Allergy Cin Immunol. (2022) 22:80–5. doi: 10.1097/ACI.0000000000000806, PMID: 35197428

[24] Xu CY Wang Q Li N Liu CH Chen YZ Shao MJ. Impact of childhood asthma on children and families and analysis of related factors in Beijing. J Environ Health. (2015) 32:10–3.

[25] Dardouri M Sahli J Ajmi T Mtiraoui A Bouguila J Zedini C. Effect of family empowerment education on pulmonary function and quality of life of children with asthma and their parents in Tunisia: a randomized controlled trial. J Pediatr Nurs. (2020) 54:e9–e16. doi: 10.1016/j.pedn.2020.04.005 32616452

[26] Jing H. Effect of relaxation training on anxiety and depression in parents of children with asthma. Nanchang: Nanchang University (2021).

[27] Chen JL Chu XL Li Y Wu LL Song FM. Effect of social support on family intimacy adaptability and depression level of parents with bronchial asthma. Maternal Child Health Care China. (2021) 36:1865–8. doi: 10.19829/j.zgfybj.issn.1001-4411.2021.08.051

[28] Guo M Gao G Guo J Wen LT Zeng LT. Burden among caregivers for children with asthma:a mixed-method study in Guangzhou, China. Inter J Nurs Sci. (2015) 2:394–401. doi: 10.1016/j.ijnss.2015.10.004

[29] Chang MH Xu SD. Analysis of the burden of primary caregivers in children with asthma and its influencing factors. Jilin Med. (2022) 43:1106–10.

[30] Li QM Zhang L. Analysis of the factors influencing the burden felt by caregivers of children with asthma. Sichuan J Physiol Sci. (2021) 43:385–7.

[31] Zhang JC Xu YC Ao ZZ Li CM Liao CF Huang YZ. Analysis of caregiver burden of children with asthma and related factors in Heyuan City. China Health Industry. (2021) 18:28–31. doi: 10.16659/j.cnki.1672-5654.2021.16.028

[32] Huang XL. Investigation on the burden and nursing knowledge demand of primary caregivers of children with asthma. Health Required Reading. (2021) 17:293.

[33] Bao YX Chen AH Fu Z Li CC Liu CH Xiang L. Guidelines for the diagnosis and prevention of asthma in children (2016edition). Chin J Pediatr. (2016) 54:167–81. doi: 10.3760/cma.j.issn.0578-1310.2016.03.003

[34] Cui Y Yang SF.Pediatric nursing. Beijing: People’s Medical Publishing House. (2017). 302–303.

[35] Li Z Liu Y. Research methods of nursing. Beijing: People's Medical Publishing House (2018). 62 p.

[36] Novak M Guest C. A comparison of the impact of institutionalization on spouse and nonspouse caregivers. J Appl Gerontol. (1992) 11:379–94. doi: 10.1177/073346489201100401, PMID: 10122828

[37] Yue P Fu Y Shang SM Liu Y Wang ZW Yu X. Reliability and validity test of caregiver burden questionnaire. Chinese J Mental Health. (2006) 8:562–4. doi: 10.3321/j.issn:1000-6729.2006.08.026

[38] Li N Chen LY Ding LY Chen XJ Yao DY Wu YY. Analysis of primary caregiver burden and its influencing factors in preschool children with scoliosis. World Latest Medical Inform Digest. (2019) 19:177–9.

[39] Qi SH Dong M. Research status of primary caregiver burden in children with chronic diseases. Tianjin Nursing. (2019) 28:244–7.

[40] Yang J Hou HR Tan JP Wang XX Xu J. Burden of family caregivers of patients with Alzheimer's disease and its influencing factors. Chinese J Clin Health. (2019) 22:170–3. doi: 10.3969/J.issn.1672-6790.2019.02.007

[41] Bamber MD Mahony H Spratling R. Mothers of children with special health care needs: Exploring caregiver burden, quality of life, and resiliency. J Pediatric Health Care. (2023). doi: 10.1016/j.pedhc.2023.06.003 37516944

[42] Zhu B. Status of quality of life in children with bronchial asthma and its relationship with family caregiver burden. Baotou Med. (2019) 44:46–9. doi: 10.3969/j.issn.1007-3507.2020.03.022

[43] Lijiao M. Interactive effects of psychological congruence and social support between children with chronic kidney disease and their carers on their quality of life. Right River College of Ethnic Medicine. (2024). doi: 10.27908/d.cnki.gymzy.2023.000127

[44] Hu JJ Wang JN Gong CY Liang H Huang QX Zhang Y. Meta-integration of care experience for family caregivers of children with asthma. Nursing Manag China. (2023) 23:79–86. doi: 10.3969/j.issn.1672-1756.2023.01.016

[45] Uzuner S Durcan G Sahin S Bahali K Barut K Kilicoglu AG. Caregiver burden and related factors in caregivers of patients with childhood-onset systemic lupus erythematosus. Clinical. (2021) 40:5025–32. doi: 10.1007/s10067-021-05867-5, PMID: 34341849

[46] Tkatch R Bazarko D Musich S Wu L MacLeod S Keown K. A pilot online mindfulness intervention to decrease caregiver burden and improve psychological well-being. J Evid Based Complementary Altern Med. (2017) 22:736–43. doi: 10.1177/2156587217737204, PMID: 29228806 PMC5871316

[47] Wang H Fan H Zhu W. Qualitative study on maternal stressors in school-age children with asthma. Modern nurse. (2022) 29:113–6. doi: 10.19792/j.cnki.1006-6411.2022.08.035

[48] Wang J Yue SJ Zhang RJ Tang YY Zhang R Guo LQ. Longitudinal study on the influence of family caregiver factors on the caring burden of patients with colorectal cancer. China Nurs Adm. (2021) 21:1250–6. doi: 10.3969/j.issn.1672-1756.2021.08.026

[49] Shangguan J Wang AM Liang PP Zhang YY Zhang YC. The relationship between family response, parents coping strategy and asthma control in children. Chin Nurs Manag. (2017) 17:1136–40. doi: 10.3969/j.issn.1672-1756.2017.08.030

[50] Yang HY Shen Q Zhang XC Wang CH. A study on the caring burden of primary caregivers in children with chronic diseases. Psychol J. (2019) 16:7–9. doi: 10.19738/j.cnki.psy.2021.19.003

[51] Ughasoro MD Eze JN Oguonu T Onwujekwe EO. Burden of childhood and adolescence asthma in Nigeria: disability adjusted life years. Paediatr Respir Rev. (2021) 41:61–67. doi: 10.1016/j.prrv.2021.07.004 34483053

[52] Abu Alrub A Hyassat D Khader YS Bani-Mustafa R Younes N Ajlouni K. Factors associated with health-related quality of life among Jordanian patients with diabetic foot ulcer. J Diabetes Res. (2019) 2019:1–8. doi: 10.1155/2019/4706720 PMC6360050 30800685

[53] Jiang D. Study on quality of life of caregivers of COPD patients with mixed methods. Jinan: Shandong University (2020).

[54] Rullander AC Lundstrom M Lindkvist M Hägglöf B Lindh V. Stress symptoms among adolescents before and after scoliosis surgery:correlations with postoperative pain. J Clin Nurs. (2016) 25:1086–94. doi: 10.1111/jocn.13137, PMID: 26898698

[55] Chen ZH Liu Y Wang R Zhou M Zhao WQ Li C. Maternal environment and drinking risk factors during pregnancy in asthma with neuropsychiatric comorbidity. Chin J Appl Clin Pediatr. (2019) 34:675–9. doi: 10.3760/cma.j.issn.2095-428X.2019.09.009

[56] Wu DM Cui WB Yu GJ. Analysis on the operation strategy of internet hospital in our country. Chinese Hospital. (2021) 25:79–80. doi: 10.19660/j.issn.1671-0592.2021.10.23

[57] Xu SD Xie J Wang Q Xu JH Chang MH. Qualitative study on the stress of primary caregivers of asthmatic children in two-child families. Modern Nurses. (2023) 30:122–5. doi: 10.19793/j.cnki.1006-6411.2023.15.030

