# Supplementary material for: Caregiver burden among parents of school-age children with asthma: a cross-sectional study
Source: Front Public Health. 2024 Jun 5;12:1368519. doi: 10.3389/fpubh.2024.1368519 (PMC11188448; doi:10.3389/fpubh.2024.1368519)
Supplement: Supplementary file 1 [file Data_Sheet_1.docx]

Version number: V1.0

Release date: January 11, 2021

**Study on parental care burden and care experience of school-age children with asthma Informed consent**

Dear subject:

We invite you to participate in the study "The Present Situation and Care Experience of Parents of school-age children with Asthma" led by Deyang City People's Hospital. This study will be carried out in Deyang People's Hospital, The Ethical Committee of Southwest Medical University Affiliated Hospital and The Ethical Committee of North Sichuan Medical College Affiliated . This study was reviewed and approved by the hospital Ethics Committee.

1.Why was this study conducted?

The prevalence of asthma in school-age children is high and the harm is great. The care of children with asthma is a long-term process, and the enthusiasm of parents to take care of children is an important factor to determine the prognosis of children with asthma. However, at present, there are few relevant studies on the parental care burden of children with asthma, and the coping strategies for the care burden are also relatively scarce. Therefore, it is of great significance to raise attention to the parental care burden of children with asthma. The aim of this study was to (1) investigate the present situation of parents' caring burden of school-age children with asthma; (2) To analyze the factors affecting the parental care burden of school-age children with asthma; (3) To understand the real experience in the process of parental care for school-age children with asthma, and propose corresponding countermeasures, so as to provide theoretical and practical guidance for alleviating the burden of parental care for children with asthma.

2.What do you need to do if you participate in a study?

If you are selected for the first part of the study, you will need to spend about 10 to 15 minutes to fill out the two questionnaires, and if you are selected for the second part of the study, you will spend 30 to 50 minutes to answer the questions based on the interview outline we have prepared.

3. What treatment options are available?

You can participate in other relevant status research questionnaires or psychological surveys as needed,don*'*t affect your participation in this study.

4. Who should not participate in the study?

If you are a child with an organic disease, a mental illness, or a serious chronic disease other than asthma; ② You have a mental illness, a serious chronic illness, and are unable to complete the questionnaire/interviewer independently.

5. What are the risks of participating in a study?

The research follows strict confidentiality and the principle of no harm, and will not cause adverse effects on you and your family.

6. What are the possible benefits of participating in a study?

By participating in this study, you will inform your real situation, which will not only help the medical staff to accurately assess your care burden, help you get positive feelings and social support, and promote the disease management of the children, but also help the nursing staff to put forward targeted coping strategies, so as to reduce your care burden more effectively and accurately. In addition, It can also provide data support for future research on children with asthma, and help to further improve the prevention and management level of childhood asthma in China.

7. Do I have to pay for participating in the study?

There is no fee to participate in this study, and there will be no fee waiver.

8. Is personal information confidential?

The information collected in this study will be completely confidential, untraceable and will not cause adverse effects to you or the child.

9. Do I have to participate in the study?

Participation in the study was completely voluntary.

**Subject Declaration:** I have read the above introduction to this study, my researcher has fully explained and explained to me the purpose of this study, the procedure, the possible risks and potential benefits of participating in this study, and answered all my relevant questions. Volunteer for this study.

I agree □ refuse□ to use my research data and biological specimens for research other than this study.

Subject's block name:

Subject signature: Date: Y M D

Subject's contact number: Mobile number:

**Investigator Statement:** I have explained the details of the study to the above-mentioned volunteer and provided him/her with an original signed informed consent form. I confirm that subjects have been explained in detail about the study, in particular the ethical principles and requirements of participating in the study, such as risks and benefits, free of charge and compensation, damages and compensation, voluntarianism and confidentiality.

Investigator Signature: Date: Y M D

Researcher contact number:

Ethics Committee of Deyang People’s Hospital

Tel: 0838-2312773 0838-2418826
