# Supplementary material for: Caregiver burden among parents of school-age children with asthma: a cross-sectional study
Source: Front Public Health. 2024 Jun 5;12:1368519. doi: 10.3389/fpubh.2024.1368519 (PMC11188448; doi:10.3389/fpubh.2024.1368519)
Supplement: Supplementary file 2 [file Data_Sheet_2.docx]

Version number: V1.0

Release date: January 11, 2021

**Caregivers Burden Inventory**

There are 24 questions below, please read each question carefully, according to your actual situation in the last week to choose the appropriate option.

1. Most of the patient's daily life needs my assistance

A never B occasionally C sometimes D often E always

2. Patients are very dependent on me

A never B occasionally C sometimes D often E always

3. I have to take care of patients all the time

A never B occasionally C sometimes D often E always

4. I have to help patients with many basic activities

A never B occasionally C sometimes D often E always

5. I don't get a moment's rest while taking care of patients

A never B occasionally C sometimes D often E always

6. I feel unable to enjoy my life

A never B occasionally C sometimes D often E always

7. I want to get out of my present situation in life

A never B occasionally C sometimes D often E always

8. My social life has suffered

A never B occasionally C sometimes D often E always

9. I am exhausted from taking care of patients

A never B occasionally C sometimes D often E always

10. Life is not what I expected it to be

A never B occasionally C sometimes D often E always

11. I don't get enough sleep

A never B occasionally C sometimes D often E always

12. My health is affected

A never B occasionally C sometimes D often E always

13.Taking care of the sick makes me ill

A never B occasionally C sometimes D often E always

14. I feel weak

A never B occasionally C sometimes D often E always

15. I don't get along with my family as well as before

A never B occasionally C sometimes D often E always

16.I worked hard to take care of patients, but my family did not understand

A never B occasionally C sometimes D often E always

17. My work is not as good as what I did before

A never B occasionally C sometimes D often E always

18. I resent relatives who can help but don't

A never B occasionally C sometimes D often E always

19. The patient's behavior made me feel embarrassed

A never B occasionally C sometimes D often E always

20. I am ashamed to have such patients

A never B occasionally C sometimes D often E always

21. I hate the patients I care for

A never B occasionally C sometimes D often E always

22. I feel uncomfortable when friends come over

A never B occasionally C sometimes D often E always

23. My dealings with patients make me angry

A never B occasionally C sometimes D often E always

24. I'm having problems in my marriage

A never B occasionally C sometimes D often E always
