# Supplementary material for: Caregiver burden among parents of school-age children with asthma: a cross-sectional study
Source: Front Public Health. 2024 Jun 5;12:1368519. doi: 10.3389/fpubh.2024.1368519 (PMC11188448; doi:10.3389/fpubh.2024.1368519)
Supplement: Supplementary file 3 [file Data_Sheet_3.docx]

Version number: V1.0

Release date: January 11, 2021

**General information questionnaire**

Hello, please carefully complete the following basic information, and you think the most appropriate answer to mark "√". We will keep your information confidential, thank you for your cooperation!

**Parent general information**

1. Your gender: male female

2. Your age: (years)

3.Your highest education level: junior high school education or below high school or technical secondary school education college education master's degree or above

4. Number of children: (number)

5.Your occupation: worker farmer administrative worker service industry private business owner

6. someone smoking at hom? yes no

7. Family history of asthma: yes no

8. Family monthly income（CNY）: 3000~4999, 5000~9999,≥10000

9. Annual medical expenses of the child（CNY）: ＜3000 3000~4999 ≥5000

**General information for children**

1. Gender: boy girl
2. Age: (years)
3. Child's illness course（year): ≤1 1~2 ≥3
4. Whether the child had undergone lung function tests: yes no
5. Number of emergency visits due to asthma exacerbation in the past three months（time）:
6. Whether the child had missed school due to asthma exacerbation in the past three months:yes no
