# Supplementary material for: Caregiver burden among parents of school-age children with asthma: a cross-sectional study
Source: Front Public Health. 2024 Jun 5;12:1368519. doi: 10.3389/fpubh.2024.1368519 (PMC11188448; doi:10.3389/fpubh.2024.1368519)
Supplement: Supplementary file 4 [file Table_1.docx]

**Supplements 1：Univariate analysis results**

**1.Comparison of caregiver burden scores in parents of children with different characteristics of school-age asthma(n =366)**

| Item | N | Constituent Ratio（%） | Time-dependence | | | Developmental | | | Physical | | | Social | | | | Emotional | | | Total caregiver burden | | | | |  |
| --- | --- | --- | --- | --- | --- | --- | --- | --- | --- | --- | --- | --- | --- | --- | --- | --- | --- | --- | --- | --- | --- | --- | --- | --- |
|  |  |  | M（P25,P75） | Z/*$\chi^{2}$* | *p* | M（P25,P75） | Z/*$\chi^{2}$* | *p* | M（P25, P75） | Z/*$\chi^{2}$* | *p* | M（P25,P75） | Z/*$\chi^{2}$* | *p* | | M（P25,P75） | Z/*$\chi^{2}$* | *p* | M（P25,P75） | | Z/*$\chi^{2}$* | *p* | |  |
| **parents' gender** |  |  |  |  |  |  |  |  |  |  |  |  | | | |  | | |  | | | | |  |
| male | 79 | 21.6 | 7(5,11) | 3.913 | <0.001 | 6(3,10) | 1.908 | 0.056 | 4(1,8) | 2.063 | 0.039 | 2(0,6) | 1.200 | 0.230 | | 1(0,5) | 0.341 | 0.733 | 21(13,39) | | 2.405 | 0.016 | |  |
| female | 287 | 78.4 | 10(6,14) |  |  | 7(5,11) |  |  | 5(3,8) |  |  | 3(1,6) |  |  | | 1(0,4) |  |  | 28(18,29) | |  |  | |  |
| **parents' age（year）** |  |  |  |  |  |  |  |  |  |  |  |  | | | |  | | |  | | | | |  |
| <30 | 48 | 13.1 | 10(7,14) | 1.254 | 0.534 | 6(4,13) | 0.385 | 0.825 | 5(2,8) | 0.145 | 0.930 | 3(1,8) | 2.002 | 0.368 | | 1(0,6) | 2.467 | 0.491 | 25(17,46) | 0.857 | | | 0.651 | |
| 30~ | 259 | 70.8 | 10(6,13) |  |  | 7(5,10) |  |  | 4(3,8) |  |  | 4(1,6) |  | |  | 1(0,5) |  |  | 28(17,38) | |  | |  |  |
| ≥40 | 59 | 16.1 | 8(6,13) |  |  | 7(3,11) |  |  | 5(2,8) |  |  | 2(0,6) |  | |  | 0(0,4) |  |  | 24(15,39) | |  | |  |  |
| **parents' highest education level** |  |  |  |  |  |  |  |  |  |  |  |  |  | |  |  |  |  |  | |  | |  |  |
| junior high school education or below | 53 | 14.4 | 8(5,13) | 0.728 | 0.867 | 8(3,12) | 4.454 | 0.216 | 5(2,7) | 9.083 | 0.028 | 3(1,6) | 5.668 | | 0.129 | 1(0,3) | 6.007 | 0.111 | 27(14,39) | | 5.835 | | 0.120 |  |
| high school or technical secondary school education | 113 | 30.9 | 10(6,14) |  |  | 7(5,11) |  |  | 5(2,8) |  |  | 3(0,6) |  |  | | 1(0,6) |  |  | 30(16,42) | |  | |  |  |
| college education | 118 | 32.2 | 9(6,13) |  |  | 6(3,10) |  |  | 4(2,7) |  |  | 3(0,6) |  |  | | 1(0,3) |  |  | 24(15,35) | |  | |  |  |
| master's degree or above | 82 | 22.5 | 9(6,12) |  |  | 7(5,10) |  |  | 6(4,8) |  |  | 4(2,7) |  |  | | 2(0,5) |  |  | 30(21,41) | |  | |  |  |
| **number of children** |  |  |  |  |  |  |  |  |  |  |  |  |  |  | |  |  |  |  | |  | |  |  |
| 1 child | 233 | 63.7 | 10(6,13) | 1.693 | 0.090 | 7(4,11) | 0.313 | 0.755 | 4(2,8) | 0.266 | 0.790 | 3(1,7) | 1.024 | 0.306 | | 1(0,5) | 1.661 | 0.097 | 28(17,41) | | 1.069 | | 0.285 |  |
| 2 or more children | 133 | 36.3 | 9(5,12) |  |  | 7(5,10) |  |  | 5(3,8) |  |  | 3(1,6) |  |  | | 1(0,3) |  |  | 27(17,36) | |  | |  |  |
| **occupation** |  |  |  |  |  |  |  |  |  |  |  |  |  |  | |  |  |  |  | |  | |  |  |
| worker | 52 | 14.2 | 7(5,11) | 9.273 | 0.055 | 6(3,9) | 6.252 | 0.181 | 4(2,6) | 8.921 | 0.063 | 2(0,4) | 9.948 | 0.041 | | 1(0,3) | 3.415 | 0.491 | 22(14,32) | | 9.904 | | 0.042 |  |
| farmer | 63 | 17.2 | 8(5,12) |  |  | 6(4,11) |  |  | 4(1,6) |  |  | 2(0,6) |  |  | | 1(0,5) |  |  | 24(13,37) | |  | |  |  |
| administrative worker | 44 | 12.0 | 11(7,13) |  |  | 8(6,9) |  |  | 6(4,9) |  |  | 3(1,6) |  |  | | 1(0,3) |  |  | 29(21,38) | |  | |  |  |
| service industry | 129 | 35.2 | 10(6,14) |  |  | 7(4,11) |  |  | 5(3,8) |  |  | 4(1,7) |  |  | | 1(0,5) |  |  | 28(18,43) | |  | |  |  |
| private business owner | 78 | 21.4 | 10(7,14) |  |  | 7(5,11) |  |  | 5(4,8) |  |  | 4(1,6) |  |  | | 2(0,6) |  |  | 30(19,43) | |  | |  |  |
| **someone smoking at home** |  |  |  |  |  |  |  |  |  |  |  |  |  |  | |  |  |  |  | |  | |  |  |
| yes | 199 | 54.4 | 9(6,13) | 0.428 | 0.669 | 6(4,11) | 0.847 | 0.397 | 0.397 | 0.737 | 0.461 | 3(1,6) | 0.172 | 0.864 | | 1(0,5) | 0.895 | 0.371 | 27(16,39) | | 0.632 | | 0.527 |  |
| no | 167 | 45.6 | 9(6,13) |  |  | 7(5,11) |  |  | 0.397 |  |  | 3(1,6) |  |  | | 1(0,4) |  |  | 28(17,39) | |  | |  |  |
| **family history of asthma** |  |  |  |  |  |  |  |  |  |  |  |  |  |  | |  |  |  |  | |  | |  |  |
| yes | 55 | 15.0 | 8(6,12) | 1.433 | 0.152 | 5(3,10) | 1.689 | 0.091 | 4(2,7) | 0.894 | 0.371 | 2(0,6) | 1.398 | 0.162 | | 1(0,2) | 2.520 | 0.012 | 21(13,33) | | 1.984 | | 0.047 |  |
| no | 311 | 85.0 | 10(6,13) |  |  | 7(5,11) |  |  | 5(3,8) |  |  | 3(1,6) |  |  | | 2(0,5) |  |  | 28(17,40) | |  | |  |  |
| **monthly family income（CNY）** |  |  |  |  |  |  |  |  |  |  |  |  |  |  | |  |  |  |  | |  | |  |  |
| 3000~4999 | 128 | 35.0 | 9(6,13) | 0.142 | 0.931 | 7(4,11) | 1.419 | 0.492 | 4(2,8) | 6.984 | 0.030 | 3(0,7) | 0.709 | 0.701 | | 1(0,5) | 1.991 | 0.370 | 27(15,40) | | 1.233 | | 0.540 |  |
| 5000~9999 | 142 | 38.8 | 10(6,14) |  |  | 7(4,11) |  |  | 4(2,7) |  |  | 3(1,5) |  |  | | 1(0,5) |  |  | 27(16,38) | |  | |  |  |
| ≥10000 | 96 | 26.2 | 9(6,12) |  |  | 7(5,10) |  |  | 6(4,9) |  |  | 3(1,6) |  |  | | 2(0,4) |  |  | 28(19,41) | |  | |  |  |
| **annual medical expenses of the child（CNY）** |  |  |  |  |  |  |  |  |  |  |  |  |  |  | |  |  |  |  | |  | |  |  |
| ＜3000 | 122 | 33.3 | 8(5,12) | 4.163 | 0.125 | 5(3,10) | 12.920 | 0.002 | 4(1,7) | 11.850 | 0.003 | 3(0,5) | 3.397 | 0.183 | | 1(0,5) | 1.876 | 0.391 | 23(13,35) | | 10.280 | | 0.006 |  |
| 3000~4999 | 154 | 42.1 | 10(6,13) |  |  | 7(5,11) |  |  | 5(3,8) |  |  | 4(0,6) |  |  | | 1(0,5) |  |  | 28(17,41) | |  | |  |  |
| ≥5000 | 90 | 24.6 | 10(7,14) |  |  | 9(5,12) |  |  | 5(3,9) |  |  | 4(1,6) |  |  | | 2(0,4) |  |  | 32(19,42) | |  | |  |  |

**2.Comparison of caregiver burden scores in children with different characteristics (n =366)**

| Item | N | Constituent Ratio（%） | Time-dependence | | | Developmental | | | Physical | | | Social | | | | | Emotional | | | | Total caregiver burden | | |
| --- | --- | --- | --- | --- | --- | --- | --- | --- | --- | --- | --- | --- | --- | --- | --- | --- | --- | --- | --- | --- | --- | --- | --- |
|  |  |  | M（P25,P75） | Z/*$\chi^{2}$* | *p* | M（P25,P75） | Z/*$\chi^{2}$* | *p* | M（P25,P75） | Z/*$\chi^{2}$* | *p* | M（P25,P75） | | Z/*$\chi^{2}$* | | *p* | M（P25,P75） | | Z/*$\chi^{2}$* | *p* | M（P25,P75） | Z/*$\chi^{2}$* | *p* |
| **child's gender** |  |  |  |  |  |  |  |  |  |  |  |  | | | | |  | | | |  | | |
| boy | 79 | 21.6 | 9(5,13) | 1.797 | 1.797 | 7(4,11) | 0.690 | 0.490 | 4(2,8) | 0.431 | 0.667 | 3(1,6) | | 1.167 | | 0.243 | 1(0,4) | | 1.637 | 0.102 | 26(16,38) | 1.530 | 0.126 |
| girl | 287 | 78.4 | 10(6,14) |  |  | 7(5,10) |  |  | 5(3,8) |  |  | 4(1,7) | |  | |  | 2(0,6) | |  |  | 29(18,42) |  |  |
| **child's age（year）** |  |  |  |  |  |  |  |  |  |  |  |  | | | | |  | | | |  | | |
| 6~ | 121 | 33.1 | 10(6,13) | 2.638 | 0.451 | 7(3,11) | 1.422 | 0.700 | 5(2,8) | 2.316 | 0.509 | 4(1,6) | 0.306 | | 0.959 | | 1(0,3) | 0.796 | | 0.850 | 27(17,38) | 0.689 | 0.876 |
| 7~ | 94 | 25.7 | 10(6,14) |  |  | 8(4,11) |  |  | 5(3,8) |  |  | 3(1,6) |  | |  | | 1(0,5) |  | |  | 29(16,43) |  |  |
| 8~ | 73 | 19.9 | 9(6,13) |  |  | 7(5,10) |  |  | 4(3,9) |  |  | 4(1,6) |  | |  | | 1(0,5) |  | |  | 28(19,41) |  |  |
| ≥9 | 78 | 21.3 | 8(5,12) |  |  | 6(4,10) |  |  | 4(2,7) |  |  | 3(1,6) |  | |  | | 2(0,6) |  | |  | 27(15,37) |  |  |
| **child's illness course（year）** |  |  |  |  |  |  |  |  |  |  |  |  |  | |  | |  |  | |  |  |  |  |
| ≤1 | 144 | 39.3 | 10(6,13) | 1.218 | 0.544 | 7(4,11) | 0.145 | 0.930 | 4(3,8) | 3.432 | 0.180 | 3(1,6) | 0.027 | | 0.986 | | 1(0,5) | 1.747 | | 0.417 | 29(17,38) | 0.090 | 0.956 |
| 1~2 | 141 | 38.5 | 9(6,13) |  |  | 7(4,10) |  |  | 4(2,8) |  |  | 3(1,6) |  | |  | | 1(0,5) |  | |  | 26(16,40) |  |  |
| ≥3 | 81 | 22.1 | 8(6,13) |  |  | 7(5,11) |  |  | 6(2,9) |  |  | 3(1,7) |  | |  | | 1(0,3) |  | |  | 27(17,39) |  |  |
| **whether the child had undergone lung function tests** |  |  |  |  |  |  |  |  |  |  |  |  |  | |  | |  |  | |  |  |  |  |
| yes | 340 | 92.9 | 9(6,13) | 0.133 | 0.894 | 7(4,11) | 0.221 | 0.825 | 5(2,8) | 0.242 | 0.809 | 3(1,6) | 0.912 | | 0.362 | | 1(0,4) | 2.122 | | 0.034 | 27(17,39) | 0.722 | 0.470 |
| no | 26 | 7.1 | 11(5,14) |  |  | 9(4,11) |  |  | 5(1,9) |  |  | 4(1,7) |  | |  | | 3(0,7) |  | |  | 33(16,47) |  |  |
| **number of emergency visits due to asthma exacerbation in the past three months（time）** |  |  |  |  |  |  |  |  |  |  |  |  |  | |  | |  |  | |  |  |  |  |
| 0 | 112 | 30.6 | 7(5,11) | 21.839 | 0.000 | 5(2,9) | 18.365 | 0.000 | 4(1,6) | 23.271 | 0.000 | 1(0,4) | 26.905 | | 0.000 | | 0(0,2) | 22.378 | | 0.000 | 20(13,31) | 33.019 | 0.000 |
| 1 | 95 | 26.0 | 10(6,13) |  |  | 8(5,11) |  |  | 5(3,8) |  |  | 4(1,6) |  | |  | | 1(0,4) |  | |  | 29(20,38) |  |  |
| 2 | 69 | 18.9 | 10(6,14) |  |  | 8(5,11) |  |  | 5(4,9) |  |  | 4(1,7) |  | |  | | 2(0,5) |  | |  | 30(19,43) |  |  |
| ≥3 | 90 | 24.5 | 11(8,14) |  |  | 8(5,13) |  |  | 6(4,9) |  |  | 4(2,8) |  | |  | | 3(0,7) |  | |  | 35(21,48) |  |  |
| **whether the child had missed school due to asthma exacerbation in the past three months** |  |  |  |  |  |  |  |  |  |  |  |  |  | |  | |  |  | |  |  |  |  |
| no | 198 | 54.1 | 8(5,12) | 4.085 | 0.001 | 6(3,10) | 2.698 | 0.007 | 4(2,8) | 3.626 | 0.000 | 2(0,5) | 3.705 | | 0.000 | | 1(0,3) | 4.345 | | 0.000 | 25(13,36) | 4.308 | 0.000 |
| yes | 168 | 45.9 | 11(7,14) |  |  | 8(5,11) |  |  | 5(4,8) |  |  | 4(1,7) |  | |  | | 2(0,6) |  | |  | 32(21,44) |  |  |

**Supplements 2：Multiple linear regression analysis of caregiver burden**

| **Dependent variable** | **Independent variable** | ***B*** | ***SE*** | ***Wald*** | ***P*** | ***OR*** | **95%*CI*** | $\chi^{2}$ | P |
| --- | --- | --- | --- | --- | --- | --- | --- | --- | --- |
| **Caregiver burden** | **Model1** |  |  |  |  |  |  | 41.249 | <0.001 |
|  | **occupation** |  |  | 11.217 | 0.024 |  |  |  |  |
|  | worker |  |  |  |  | 1 |  |  |  |
|  | farmer | 0.999 | 0.504 | 3.924 | 0.048 | 2.715 | 1.011~7.293 |  |  |
|  | administrative worker | 0.927 | 0.541 | 2.937 | 0.087 | 2.527 | 0.875~7.296 |  |  |
|  | service industry | 1.442 | 0.461 | 9.768 | 0.002 | 4.229 | 1.712~10.446 |  |  |
|  | private business owner | 1.369 | 0.485 | 7.975 | 0.005 | 3.933 | 1.520~10.174 |  |  |
|  | **annual medical expenses of the child（CNY）** |  |  | 6.878 | 0.032 |  |  |  |  |
|  | ＜3000 |  |  |  |  | 1 |  |  |  |
|  | 3000~4999 | 0.601 | 0.293 | 4.220 | 0.040 | 1.824 | 1.028~3.237 |  |  |
|  | ≥5000 | 0.802 | 0.321 | 6.237 | 0.013 | 2.230 | 1.188~4.184 |  |  |
|  | **number of emergency visits due to asthma exacerbation in the past three months（time）** |  |  | 17.059 | 0.001 |  |  |  |  |
|  | 0 |  |  |  |  | 1 |  |  |  |
|  | 1 | 1.104 | 0.347 | 10.117 | 0.001 | 3.018 | 1.528~.959 |  |  |
|  | 2 | 1.052 | 0.367 | 8.241 | 0.004 | 2.865 | 1.396~5.877 |  |  |
|  | ≥3 | 1.352 | 0.343 | 15.526 | 0.000 | 3.867 | 1.973~7.577 |  |  |
|  | **Model2** |  |  |  |  |  |  | 30.970 | <0.001 |
|  | **occupation** |  |  | 11.487 | 0.022 |  |  |  |  |
|  | worker |  |  |  |  | 1 |  |  |  |
|  | farmer | 0.649 | 0.636 | 1.041 | 0.308 | 1.914 | 0.550~6.661 |  |  |
|  | administrative worker | 0.219 | 0.718 | 0.093 | 0.760 | 1.245 | 0.305~5.083 |  |  |
|  | service industry | 1.369 | 0.568 | 5.819 | 0.016 | 3.932 | 1.293~11.959 |  |  |
|  | private business owner | 1.305 | 0.591 | 4.876 | 0.027 | 3.687 | 1.158~11.740 |  |  |
|  | **number of emergency visits due to asthma exacerbation in the past three months（time）** |  |  | 17.122 | 0.001 |  |  |  |  |
|  | 0 |  |  |  |  | 1 |  |  |  |
|  | 1 | 0.615 | 0.431 | 2.041 | 0.153 | 1.850 | 0.795~4.305 |  |  |
|  | 2 | 1.142 | 0.426 | 7.171 | 0.007 | 3.133 | 1.358~7.226 |  |  |
|  | ≥3 | 1.544 | 0.396 | 15.186 | 0.000 | 4.684 | 2.154~10.183 |  |  |
| **Time-dependenceburden** | **Model1** |  |  |  |  |  |  | 18.742 | <0.001 |
|  | **parents' gender** | 0.984 | 0.308 | 10.223 | 0.001 | 2.674 | 1.463~4.887 |  |  |
|  | **whether the child had missed school due to asthma exacerbation in the past three months** | 0.907 | 0.229 | 15.714 | 0.000 | 2.478 | 1.582~3.881 |  |  |
|  | **Model2** |  |  |  |  |  |  | 9.872 | <0.001 |
|  | **parents' gender** | 1.267 | 0.400 | 10.033 | 0.002 | 3.551 | 1.621~7.779 |  |  |
|  | **whether the child had missed school due to asthma exacerbation in the past three months** | 0.647 | 0.255 | 6.438 | 0.011 | 1.910 | 1.159~3.149 |  |  |
| **Developmental**  **burden** | **Model1** |  |  |  |  |  |  | 10.637 | 0.014 |
|  | **number of emergency visits due to asthma exacerbation in the past three months（time）** |  |  | 13.629 | 0.003 |  |  |  |  |
|  | 0 |  |  |  |  | 1 |  |  |  |
|  | 1 | 0.959 | 0.318 | 9.125 | 0.003 | 2.610 | 1.401~4.863 |  |  |
|  | 2 | 0.967 | 0.343 | 7.963 | 0.005 | 2.630 | 1.344~5.148 |  |  |
|  | ≥3 | 1.049 | 0.320 | 10.748 | 0.001 | 2.856 | 1.525~5.348 |  |  |
|  | **Model2** |  |  |  |  |  |  | 15.716 | 0.001 |
|  | **number of emergency visits due to asthma exacerbation in the past three months（time）** |  |  | 11.879 | 0.008 |  |  |  |  |
|  | 0 |  |  |  |  |  |  |  |  |
|  | 1 | 0.272 | 0.401 | 0.460 | 0.498 | 1.312 | 0.598~2.880 |  |  |
|  | 2 | 0.748 | 0.404 | 3.434 | 0.064 | 2.113 | 0.958~4.662 |  |  |
|  | ≥3 | 1.151 | 0.365 | 9.925 | 0.002 | 3.161 | 1.545~6.469 |  |  |
| **Physical**  **burden** | **Model1** |  |  |  |  |  |  | 45.814 | <0.001 |
|  | **number of emergency visits due to asthma exacerbation in the past three months（time）** |  |  | 10.003 | 0.019 |  |  |  |  |
|  | 0 |  |  |  |  | 1 |  |  |  |
|  | 1 | 0.770 | 0.345 | 4.962 | 0.026 | 2.159 | 1.097~4.249 |  |  |
|  | 2 | 0.930 | 0.371 | 6.284 | 0.012 | 2.536 | 1.225~5.248 |  |  |
|  | ≥3 | 1.002 | 0.345 | 8.445 | 0.004 | 2.724 | 1.386~5.355 |  |  |
|  | **occupation** |  |  | 12.681 | 0.013 |  |  |  |  |
|  | worker |  |  |  |  | 1 |  |  |  |
|  | farmer | -0.197 | 0.512 | 0.148 | 0.701 | 0.821 | 0.301~2.241 |  |  |
|  | administrative worker | 1.148 | 0.507 | 5.128 | 0.024 | 3.152 | 1.167~8.513 |  |  |
|  | service industry | 0.904 | 0.435 | 4.307 | 0.038 | 2.468 | 1.052~5.795 |  |  |
|  | private business owner | 0.960 | 0.475 | 4.086 | 0.043 | 2.612 | 1.030~6.624 |  |  |
|  | **monthly family income（CNY）** |  |  | 10.147 | 0.006 |  |  |  |  |
|  | 3000~4999 |  |  |  |  | 1 |  |  |  |
|  | 5000~9999 | -0.899 | 0.321 | 7.822 | 0.005 | 0.407 | 0.217~0.764 |  |  |
|  | ≥10000 | -0.081 | 0.340 | 0.057 | 0.811 | 0.922 | 0.474~1.795 |  |  |
|  | **annual medical expenses of the child（CNY）** |  |  | 7.364 | 0.025 |  |  |  |  |
|  | ＜3000 |  |  |  |  | 1 |  |  |  |
|  | 3000~4999 | 0.686 | 0.313 | 4.798 | 0.028 | 1.986 | 1.075~3.668 |  |  |
|  | ≥5000 | 0.884 | 0.342 | 6.678 | 0.010 | 2.422 | 1.238~4.736 |  |  |
|  | **Model2** |  |  |  |  |  |  | 14.801 | <0.001 |
|  | **number of emergency visits due to asthma exacerbation in the past three months（time）** |  |  | 10.007 | 0.019 |  |  |  |  |
|  | 0 |  |  |  |  | 1 |  |  |  |
|  | 1 | 0.782 | 0.418 | 3.503 | 0.061 | 2.186 | 0.964~4.957 |  |  |
|  | 2 | 1.184 | 0.431 | 7.536 | 0.006 | 3.269 | 1.403~7.616 |  |  |
|  | ≥3 | 1.171 | 0.406 | 8.328 | 0.004 | 3.226 | 1.456~7.145 |  |  |
|  | **monthly family income（CNY）** |  |  | 8.694 | 0.013 |  |  |  |  |
|  | 3000~4999 |  |  |  |  | 1 |  |  |  |
|  | 5000~9999 | -0.788 | 0.348 | 5.125 | 0.024 | 0.455 | 0.230~0.900 |  |  |
|  | ≥10000 | 0.201 | 0.337 | 0.354 | 0.552 | 1.222 | 0.631~2.367 |  |  |
|  | **annual medical expenses of the child（CNY）** |  |  | 8.510 | 0.014 |  |  |  |  |
|  | ＜3000 |  |  |  |  | 1 |  |  |  |
|  | 3000~4999 | 0.758 | 0.368 | 4.237 | 0.040 | 2.133 | 1.037~4.390 |  |  |
|  | ≥5000 | 1.133 | 0.390 | 8.458 | 0.004 | 3.105 | 1.447~6.663 |  |  |
| **Social**  **burden** | **Model1** |  |  |  |  |  |  | 24.279 | <0.001 |
|  | **whether the child had missed school due to asthma exacerbation in the past three months** | 0.698 | 0.225 | 9.576 | 0.002 | 2.009 | 1.292~3.126 |  |  |
|  | **occupation** |  |  | 11.526 | 0.021 |  |  |  |  |
|  | worker |  |  |  |  | 1 |  |  |  |
|  | farmer | 0.387 | 0.444 | 0.758 | 0.384 | 1.472 | 0.616~3.517 |  |  |
|  | administrative worker | 0.629 | 0.471 | 1.786 | 0.181 | 1.876 | 0.746~4.719 |  |  |
|  | service industry | 1.136 | 0.388 | 8.553 | 0.003 | 3.113 | 1.454~6.663 |  |  |
|  | private business owner | 0.932 | 0.416 | 5.014 | 0.025 | 2.538 | 1.123~5.737 |  |  |
|  | **Model2** |  |  |  |  |  |  | 9.107 | 0.003 |
|  | **whether the child had missed school due to asthma exacerbation in the past three months** | 0.628 | 0.258 | 5.929 | 0.015 | 1.873 | 1.130~3.105 |  |  |
|  | **occupation** |  |  | 11.854 | 0.018 |  |  |  |  |
|  | worker |  |  |  |  | 1 |  |  |  |
|  | farmer | 0.578 | 0.543 | 1.134 | 0.287 | 1.783 | 0.615~5.167 |  |  |
|  | administrative worker | 0.155 | 0.621 | 0.062 | 0.803 | 1.168 | 0.346~3.943 |  |  |
|  | service industry | 1.279 | 0.477 | 7.176 | 0.007 | 3.593 | 1.409~9.159 |  |  |
|  | private business owner | 0.925 | 0.511 | 3.271 | 0.070 | 2.521 | 0.926~6.866 |  |  |
| **Emotional**  **burden** | **Model1** |  |  |  |  |  |  | 24.628 | <0.001 |
|  | **family history of asthma** | -1.087 | 0.376 | 8.363 | 0.004 | 0.337 | 0.161~0.705 |  |  |
|  | **whether the child had missed school due to asthma exacerbation in the past three months** | 0.947 | 0.228 | 17.286 | 0.000 | 2.578 | 1.650~4.028 |  |  |
|  | **Model2** |  |  |  |  |  |  | 12.407 | <0.001 |
|  | **whether the child had missed school due to asthma exacerbation in the past three months** | 0.875 | 0.263 | 11.070 | 0.001 | 2.400 | 1.433~4.019 |  |  |
